# 3D printing–assisted preoperative plan of pedicle screw placement for middle-upper thoracic trauma: a cohort study

**DOI:** 10.1186/s12891-017-1703-1

**Published:** 2017-08-11

**Authors:** Wei Xu, Xuming Zhang, Tie Ke, Hongru Cai, Xiang Gao

**Affiliations:** 1 Department of Emergency & Trauma Surgery, Fujian Provincial Hospital, Fuzhou, Fujian Province 350001 China; 2 Department of Trauma, Institute of Emergency Medicine, Fujian Provincial Hospital, Fuzhou, Fujian Province 350001 China; 3 Clinical Institute of Provincial Hospital, Fujian Medical University, Fuzhou, Fujian Province 350001 China

**Keywords:** 3D printing, Middle-upper thoracic trauma, Pedicle screw, Preoperative plan

## Abstract

**Background:**

This study aimed to evaluate the application of 3D printing in assisting preoperative plan of pedicle screw placement for treating middle-upper thoracic trauma.

**Methods:**

A preoperative plan was implemented in seven patients suffering from middle-upper thoracic (T3–T7) trauma between March 2013 and February 2016. In the 3D printing models, entry points of 56 pedicle screws (Magerl method) and 4 important parameters of the pedicle screws were measured, including optimal diameter (ϕ, mm), length (L, mm), inclined angle (α), head-tilting angle (+β), and tail-tilting angle (−β). In the surgery, bare-hands fixation of pedicle screws was performed using 3D printing models and the measured parameters as guidance.

**Results:**

A total of seven patients were enrolled, including five men and two women, with the age of 21–62 years (mean age of 37.7 years). The position of the pedicle screw was evaluated postoperatively using a computerized tomography scan. Totally, 56 pedicle screws were placed, including 33 pieces of level 0, 18 pieces of level 1, 4 pieces of level 2 (pierced lateral wall), and 1 piece of level 3 (pierced lateral wall, no adverse consequences), with a fine rate of 91.0%.

**Conclusions:**

3D printing technique is an intuitive and effective assistive technology to pedicle screw fixation for treating middle-upper thoracic vertebrae, which improve the accuracy of bare-hands screw placement and reduce empirical errors.

**Trial registration:**

The trial was approved by the Ethics Committee of the Fujian Provincial Hospital. It was registered on March 1st, 2013, and the registration number was K2013–03-001.

**Electronic supplementary material:**

The online version of this article (doi:10.1186/s12891-017-1703-1) contains supplementary material, which is available to authorized users.

## Background

On the basis of the barrel thoracic anatomy, thoracic vertebrae 1 to 10 are usually referred to as middle-upper thoracic vertebrae. However, it seems to lack a strict division of upper and middle thoracic vertebrae [1–4]. As the middle-upper thoracic vertebrae include 10 segments, the pedicle morphology, direction of articular facets, and volume of spinal canals differ greatly. Especially, T3–T7 is the “watershed” area [5, 6], at which the pedicle morphology differs a lot from those of the upper and lower thoracic vertebrae. Therefore, the pedicle screw placement technology and accurate grasping of pedicle morphology are crucial to surgeons managing middle-upper thoracic fracture.

3D printing, also known as rapid prototyping, is to print computerized data into 3D objects by increasing materials layer by layer using stratified manufacturing and superimposed prototyping mode. Accuracy of printing a 1:1 spine model [7] and skull model [8] by 3D printing has been confirmed. It is a readily available and inexpensive method. 3D printing have been applied in several studies, which is used for preoperative observation and individualized preoperative design, in treating upper cervical diseases [9] and spinal tumors [10]. We supposed that 3D printing technique could improve the accuracy of bare-hands screw placement and reduce empirical errors. However, not many studies are available on the application of this technique in middle-upper thoracic vertebrae. Therefore, our objective in this study is to evaluate the application of 3D printing in assisting preoperative plan of pedicle screw placement for treating middle-upper thoracic trauma. This study included patients suffering from spinal trauma at T3–T7 “watershed” middle-upper thoracic vertebrae, in which 3D printing–assisted bare-hands placement of pedicle screws in the middle-upper thoracic vertebrae was preliminarily investigated.

## Methods

### Study design and patients

In the present case series study, 13 patients with middle-upper thoracic trauma were admitted to the wards of Traumatology Department in Fujian Provincial Hospital between March 2013 and February 2016. Among those patients, conservative treatment were given to 3 stable fracture patients and 1 osteoporotic vertebral compression fractures (OVCFs) patients. Two other OVCFs patient (T5, T7 respectively) was treated with vertebroplasty. Thus 6 patients were excluded, and seven cases satisfying the inclusion criteria were selected. Patients with unstable middle-upper thoracic trauma are often concurrent with neurologic deficits, while stable fracture patients without neurologic deficits received conservative treatment. Therefore, the inclusion criteria were as follows: Patients suffering from middle-upper thoracic trauma at T3–T7 “watershed” segment accompanied with incomplete or complete spinal cord injury, and requiring surgery. The exclusion criteria were as follows: Patients with senile osteoporotic vertebral fracture and other middle-upper thoracic trauma, not requiring surgery. The final diagnosis was based on the thoracic vertebral fracture and spinal cord injury in thoracic magnetic resonance imaging.

### Reconstruction and 3D printing

**Fig. 1:**
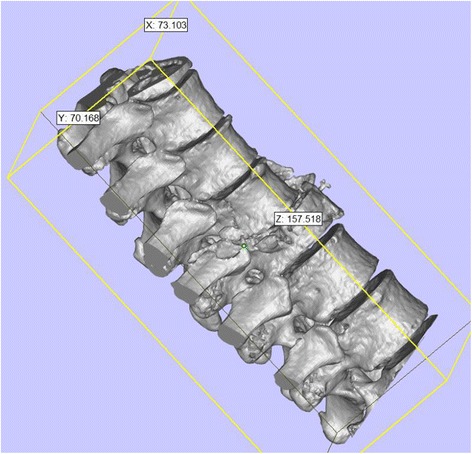
3D spine model, which can be observed from any direction on *X*, *Y*, and *Z* axes

**Fig. 2:**
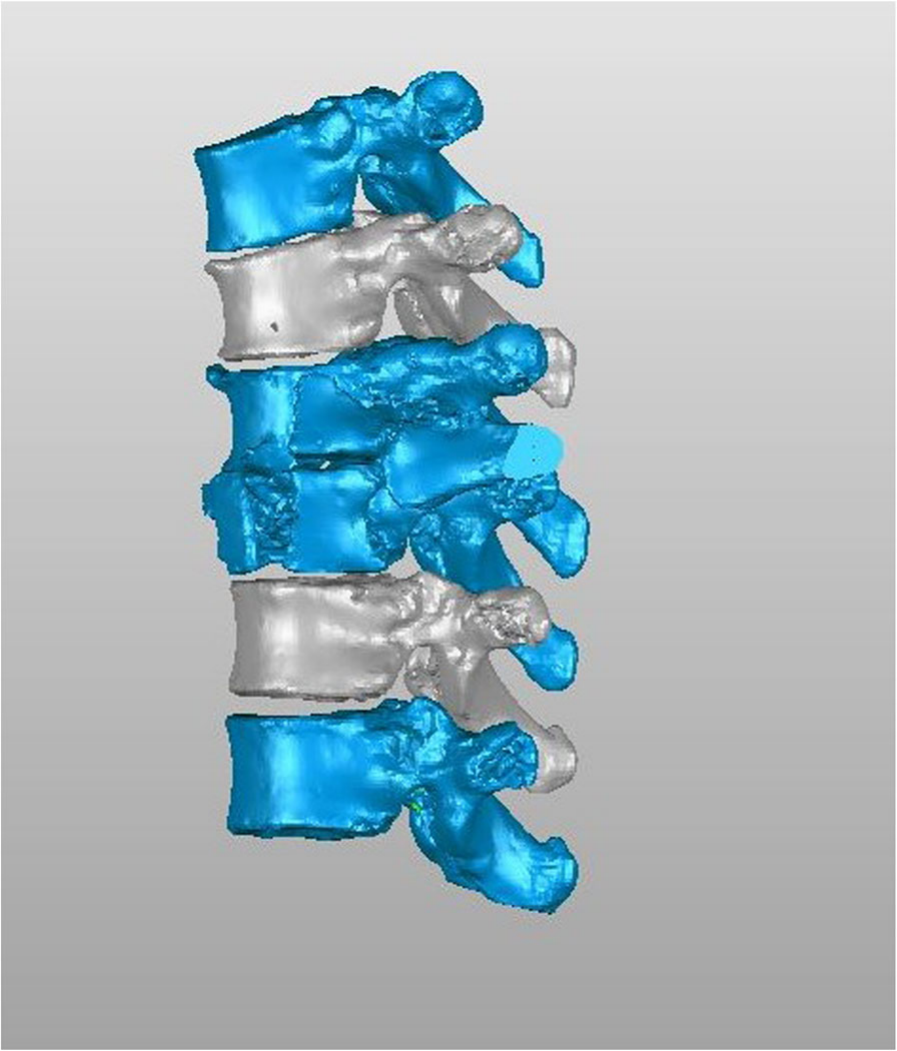
3D spine model, in which different colors can be applied for differentiation

**Fig. 3:**
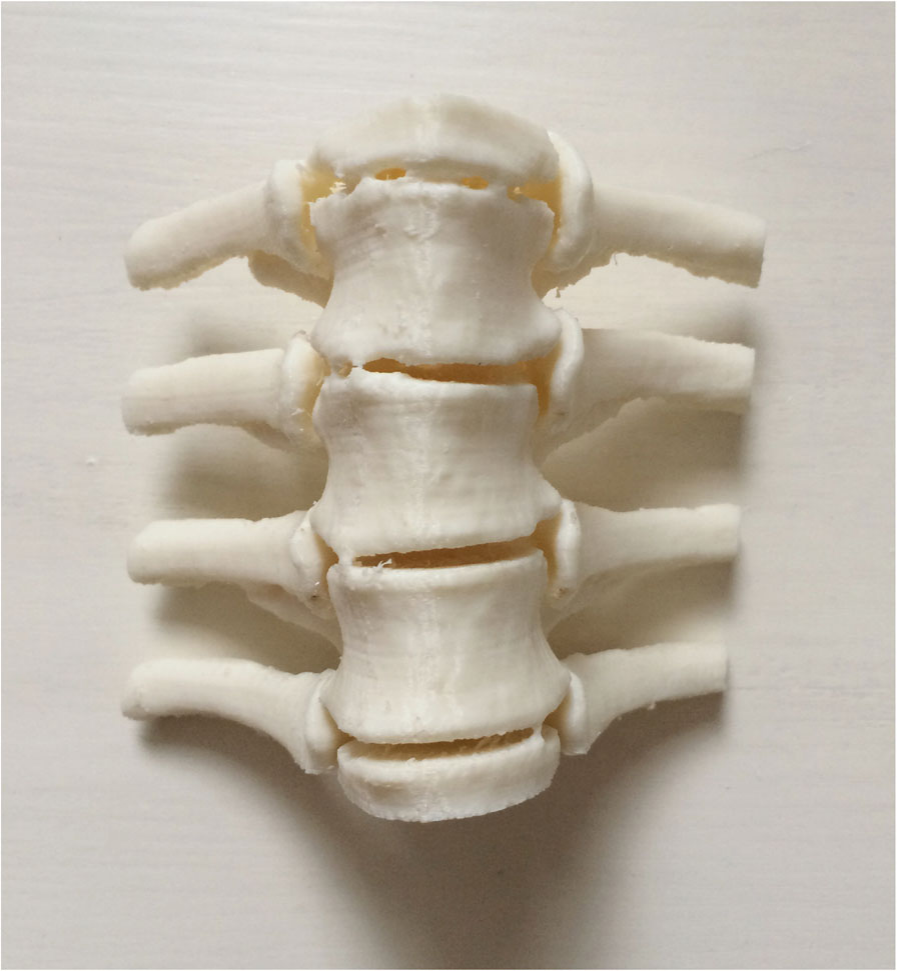
Continuously printed 3D spine model

**Fig. 4:**
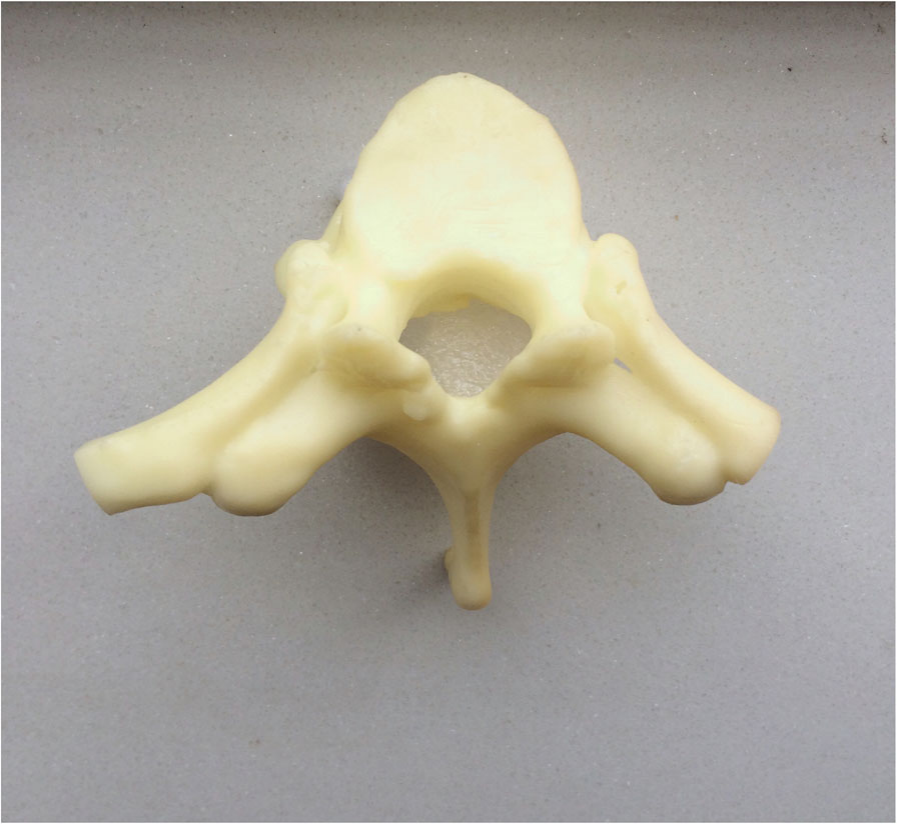
Individually printed 3D spine model

Currently, the vast majority of the computer reconstruction is performed based on the cross-sectional data (slicing data) [11]. In this study, seven patients underwent computed tomography (CT) scanning (64 CT, SOMATOM Definition, Siemens, Germany) with a slice thickness of 1.0 mm, and the raw data of each layer were stored in the Digital Imaging and Communications in Medicineformat. Then CT scan data were imported into the Mimics14.0 3D image processing software (Materialise, Belgium) for 3D reconstruction, not only to obtain a 3D fracture model, which enabled to observe the fractures from multiple angles and multiple directions (Fig. 1), but also to perform differentiation using different colors (Fig. 2). Then the reconstructed 3D fracture models were imported into the 3D printer in CAD (computer-aided design) file format, and 1:1 physical models were printed out by taking resin as the material. The models could be printed continuously (Fig. 3) or individually (Fig. 4).

For an experienced operator, the reconstruction of continuously segments would take no more than 0.5 h. Separation of each vertebra would consume nearly 1 h. The printing time was determined by the total length, which is 1.5 to 2 h for each vertebra. For example, in order to prepare a model for T1-T4, it would consume 0.5 h (total reconstruct) + 4 h (separation of each vertebra) + 2*8 h (4 continuously and 4 individually vertebra) = 20.5 h.

### Preoperative plan and screw placement simulation

**Fig. 5:**
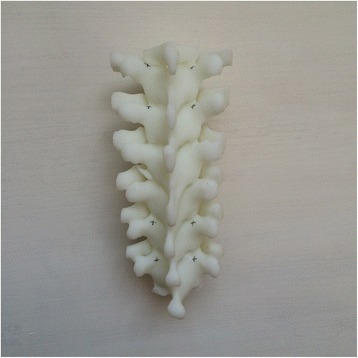
Entry points were determined using the Magerl method, and marked by *small cross shapes*

**Fig. 6:**
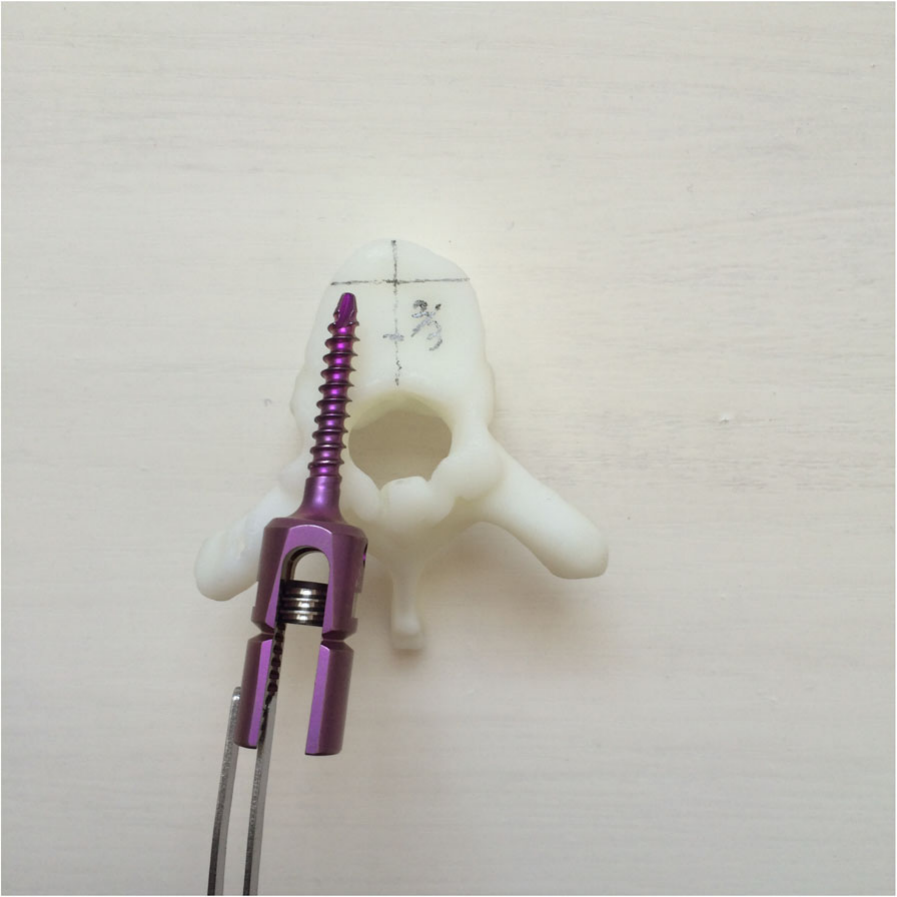
Diameter and length of the screws were selected based on the 1:1 model taking one third of the anterior vertebral body as the leading age

**Fig. 7:**
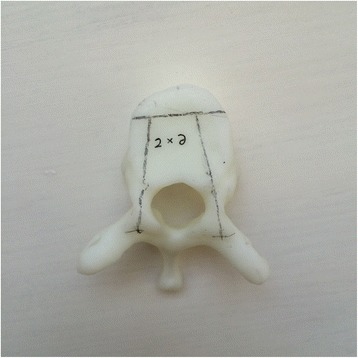
Taking one third of the anterior vertebral body as the leading age, the angle between lateral pedicles was 2 × *α*

**Fig. 8:**
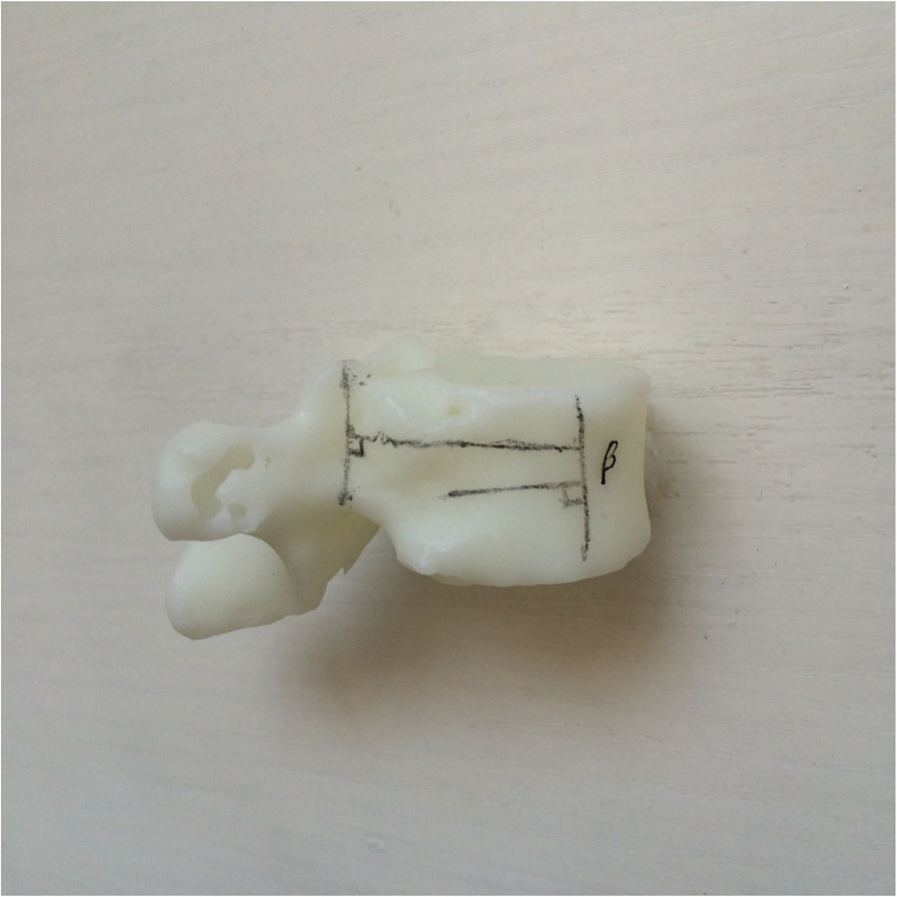
Taking one third of the anterior vertebral body as the leading age, the angle between the cross section of the pedicle and coronal plane of the vertebral body was *β*

To improve the clinical accuracy of placing pedicle screws in middle-upper thoracic vertebrae, the most important thing is to determine the entry points of pedicle screws and the four parameters (ϕ, L, α, and β): (1) In this study, the most commonly used Magerl method was considered as the standard to determine the entry points of pedicle screws on the fixed segments, which meant the intersection of the outer edge of the superior articular process and transverse horizontal midline, as shown in Fig. 5. (2) The optimal diameter (ϕ, mm) and length (L, mm) of the pedicle screws for the segment to be fixed were selected on the 1:1 printing model by considering a screw diameter that is slightly smaller than the pedicles, and one third of the front part of the vertebral body as the leading edge, as shown in Fig. 6. (3) In terms of the incline angle (α), on the 1:1 printing model, one third of the anterior vertebral body was taken as the leading edge to measure the angle between bilateral pedicles, which was 2 × α, as shown in Fig. 7. (4) In terms of the head-tilting (+β) or tail-tilting (−β) angle, one third of the anterior vertebral body was taken as the leading edge, where the angle between the cross section of the pedicle and coronal plane of the vertebral body was β, as shown in Fig. 8. It is worth noting that these four parameters can also be obtained in a professional medical image processing software, but it is significantly less intuitive and convenient compared with that obtained in the 1:1 model.

### Treatment

After the patients underwent general anesthesia, they were placed in prone position, with upper limbs held upward or drooped on both sides of the body. The diseased vertebra was positioned using X-ray fluoroscopy, a longitudinal incision of appropriate length centered at the diseased vertebra was made, and paraspinal muscles were stripped toward both sides to expose the spinous process, lamina, articular process, and part of the transverse process. At this time, the sterilized printed 3D vertebral model was put on the table. Referring to the preoperatively marked entry point, location of the vertebral entry point could be found simultaneously in the surgical vision. An incision was made, and a Kirschner wire was inserted according to the inclined angle and head-tilting (tail-tilting) angle measured preoperatively for temporary positioning. Then the wire was adjusted to the satisfactory position using anterioposterior and lateral fluoroscopy, and the solid pedicle screw was inserted followed by decompression, reduction and rod fixation, placement of a suction drainage tube, closure of the wound, and sterile dressing. No cannulated screws were used in this study. The fixed segments ranged from two vertebral body above the injured vertebral ones to two vertebral body under. Joint fusion was made between posterior small joint and interlaminar.

## Results

### Outcome measurement

In this study, a total of seven patients were icluded, comprising five men and two women, with the age of 21–62 years (mean age of 37.7 years). The regarding causes of trauma, i.e. four cases of traffic injury, two cases of falling injury, and one case of crashing object injury. Fractures were classified according Magerl classification [12]: one case of type A3, two cases of type B1, one case of B2 and three cases of type C2, of which one case had cumulative trauma at the fourth spine (fracture of the sternum and ribs). The spinal injury was classified on the basis of American Spinal Injury Association (ASIA) grading: three cases of level A, three cases of level B, and one case of level C. Detailed information of patients is listed in Table 1. The Pre-and postoperative parameters of patients were compared in Table 2.

**Table 1:** Characteristics of patients included in this study

NO	Gender	Site of injury and AO classification	Time of surgery (days after injury)	Accompanied injuries	Fixed pedicle segments
1	M	T5, type A3	3	SAH,TWL	T3-T7
2	M	T4/5, type B1	6	SAH,TWL, Stable pelvic fracture, Hematuria	T3-T6
3	M	T2/3, type B1	10	Brain bruise, Cranio-facial bones fractures, Sternum and rib fracture, Hemopneumothorax, Renal bruise, Tibiofibula fracture	T1-T4
4	M	T3/4, type B2	7	Subdural hemorrhage, Skull fracture, Clavicle fracture, Hemopneumothorax, Liver bruise	T2-T5
5	M	T4/5, type C2	2	Cranio-facial bones fractures, Rib fracture, Hemothorax	T3-T7
6	F	T7/8, type C2	3	Concussion, Cranio-facial bones fractures, Liver and renal bruise	T5-T10
7	F	T7, type C2	5	Partially stable pelvic fracture, Renal bruise, Calcaneal fracture	T5-T9

SAH Subarachnoid hemorrhage; TWL Traumatic wet lung;

**Table 2:** Pre-and postoperative parameters of patients

NO	Preoperative ASIA grading	Postoperative ASIA grading	Preoperative kyphosis	Postoperative kyphosis	Latest kyphosis	Preoperative ISS score
1	C	D	11°	7°	7°	13
2	A	A	13°	13°	14°	22
3	A	B	6°	6°	6°	36
4	A	A	7°	7°	9°	25
5	B	B	14°	14°	16°	13
6	B	B	13°	13°	14°	17
7	B	B	14°	14°	14°	17

For preoperative planning, the parameters measured from the 1:1 printed models are shown in Table 3.

**Table 3:** Measurement data

Segment	T1				T2				T3				T4				T5			
Case	*α*	*β*	*ɸ*	*L*	*α*	*β*	*ɸ*	*L*	*α*	*β*	*ɸ*	*L*	*α*	*β*	*ɸ*	*L*	*α*	*β*	*ɸ*	*L*
1									19.5	10	3.5	30	16.5	10	3.5	30				
2									23.5	18	3.5	30	21	16	3.5	30	17.5	13	4	30
3	33	14	3.5	30	30.5	13	3.5	30	24.5	11	3.5	30	19	11	3.5	30				
4					28	13	3.5	30	22.5	13	3.5	30	17.5	11	4	30	15	11	4.5	30
5									20.5	16	3.5	30	16	15	3.5	30				
6																	11.5	15	4.5	30
7																	15.5	16	4	30
Segment	T6				T7				T8				T9				T10			
Case	*α*	*β*	*ɸ*	*L*	*α*	*β*	*ɸ*	*L*	*α*	*β*	*ɸ*	*L*	*α*	*β*	*ɸ*	*L*	*α*	*β*	*ɸ*	*L*
1	13	6	4.5	35	11	4	4.5	35												
2	15	11	4.5	35																
3																				
4																				
5	11	6	4.5	35	10	5	4.5	35												
6	10	13	4.5	35									6.5	10	5	40	5	7	5.5	40
7	15	15	4.5	35					11	12	5	40	10.5	11	5	40				

*α*, the measured inclined angle of the pedicle screw (in degree); *β*, the measured head-tilting angle of the pedicle screw (in degree); *ɸ*, the optimal diameter of the pedicle screw based on the 3D printing model (in mm); *L,* the optimal length of the pedicle screw based on the 3D printing model (in mm)

The average operation time was 2 h ± 30 min. Re-replacement of Kirschner wire occurred in the first two operations. In the following cases, no re-replacement occurred, however, only slight adjustment required for the patients. The postoperative evaluation was performed after the retraction of drainage tube, which was on the third to fifth day after the surgery. Locations of pedicle screws were evaluated using X-ray and CT scanning of the posterior-anterior and lateral lumbar spine. On the basis of the currently most prevalent method for assessing the accuracy of pedicle screws [13], the penetration degree of the pedicles was graded on the CT scan images: Level 0, no penetration; level 1, penetrating to <25% of the screw diameter; level 2, penetrating to 20%–50% of the screw diameter; and level 3, penetrating to >50% of the screw diameter. From the above, levels 0 and 1 were considered excellent screw locations, while levels 2 and 3 were considered piercing the medial or lateral wall of the pedicles. In this study, a total of 56 pedicle screws were implemented, including 34 pieces of level 0, 18 pieces of level 1, 4 pieces of level 2 (piercing lateral wall), and 1 piece of level 3 (piercing lateral wall, no adverse consequences), with a screw location fine rate of 91.0%(Table 4). No postoperative infection and other complications occurred.

**Table 4:** CT evaluation results

	Level 0	Level 1	Level 2	Level 3
T1	2	0	0	0
T2 3	1	0	0	
T3	5	4	1	0
T4	4	4	2	0
T5	4	3	1	0
T6	5	4	0	0
T7	3	1	0	1
T8	2	0	0	0
T9	3	1	0	0
T10	2	0	0	0
N (Rate)	33 (58.9%)	18 (32.1%)	4 (7.2%)	1 (1.8%)
Success rate	91.0%			

### Follow-up

All the patients were followed-up for more than 6 months, with the longest duration of 3 years. Reconstruction of lumbar was successfully resulted in all patients. No severe complication occurred and all patients discharged from hospital after operations. During the follow-up, displacement, loosening, and fracture of the internal fixation were not found in the X-ray images. However, only one patients gained significant neurologic improvement and another one gained mild improvement in our follow-up. No significant neurologic improvements were reported in other five cases.

*Typical case1:* The patient was a 48-year-old female, with a traffic injury, having a blowout fracture at T7 and T8 of type C2 and ASIA level B. During the surgery, the upper wall of the right T6 pedicle was pierced accidentally, and then the pedicle screw could not be satisfactorily placed even by repeated operation. However, the model was observed and intact left pedicle was found in the diseased T7. On the basis of this model, screws with a diameter of 4.5 mm and length of 35 mm were selected and placed in the diseased vertebra, thereby achieving good efficacy. This fully embodies the advantages of 3D printing in tough cases, which enables to cope with the emergencies and promptly adjust the surgery program, as shown in Additional file 1: Figs. 9–19.

*Typical case2:* The patient was a 62-year-old male, admitted after falling, with a fracture at T2 and T3 of type B1 and ASIA level A. Continuously and sperately models were printed to assist the operation. Limited by the position, only coronal X-ray and CT scanning were available to this patient. Related imaging data including the placement of screws were shown in Additional file 2: Figs. 20–27.

## Discussion

Spinal trauma at the middle-upper thoracic vertebrae (T1–T10) is not common [14], it was reported as approximately 10%–20% of the general spinal trauma cases [15]. Trauma of this segment often caused by large external force, and often combined with multiple injuries, which frequently resulted with a poor prognosis [16, 17]. Currently, early surgical treatment is recommended for middle-upper thoracic trauma with an aim to reduce pain, stabilize spine, reconstruct sagittal and coronal sequences, and decrease mortality [18, 19].

In our study, posterior reconstruction surgery was performed after an average of 5.14 days after trauma. Among all of the patients, one patient with complete spinal nerve trauma was operated 10 days after trauma because he was a foreign worker and no immediate family was present after trauma. Due to the non-life threatening condition of the patient, we delayed the operation until the immediate family signed informed consent as legally required. Furthermore, in our operation method, we prefer the solid screws, although screw misplacement is possible despite correct K-wire positioning, due to the higher cost for cannulated screws and based on the patient’s consent.

At present, posterior approach is often implemented in surgical treatment for middle-upper thoracic trauma [20, 21]. However, thoracic pedicles are thin and have significant variations. The T1–T10 include a long span, where entry points differ at different segment and present variation [22]. Zindrick et al. found that the inclined angle of the pedicle (*α*) could be gradually decreased from 30° at T1 to 0° at T12 [23]. Studies by Vaccaro revealed that the head-tilting angle of the pedicle (+*β*) could be gradually reduced from 14° at T1 to 7° at T12 [24]. Charles et al. [25] studied 503 corpses and found that the thoracic pedicle size was strongly correlated with age, gender, height, and body weight, where it was larger in males, older, tall, and heavy-weight individuals. Deng-bin Qi et al. [26] studied the thoracic vertebra anatomy in detail in 54 adults with normal spine using 3D CT, and found that the best entry point on the thoracic vertebra constantly changed from T1 to T12, where the entry point difference between the cross-section and sagittal planes was statistically significant. Robert et al. [27] investigated the T1–T6 pedicle morphology in 18 elderly corpses, and revealed that the pedicle differed between different individuals and between different segments in the same individual, where it was narrowest at the T4–T6, and the secure pedicle screw length changed from 30 mm at T1 to 40 mm at T6.

Thoracic surgery demands more stringent requirements are followed on pedicle screw placement. In the traditional posterior approach, the accuracy of the pedicle screws is determined by open placement technique and intraoperative two-dimensional fluoroscopy, which still shows a high pedicle screw error rate.

FeyzaKaragoz et al. conducted a cohort study including 113 T2–T8 pedicle screws in 24 patients with absence of coronal deformity by means of intraoperative single C-arm fluoroscopy and postoperative CT evaluation, and found a screw placement error rate of 20.3%, where it was 27.4% at T2–T5 and 14.5% at T6–T8. In a study conducted by Vaccaro et al. [28], bare-hands placement of pedicle screws was performed on T4–T12 of five fresh corpses by five experienced spinal surgeons, followed by postoperative CT assessment, and their results showed a placement error rate up to 41%, of which 21 screws penetrated the medial wall of the pedicles and entered the vertebral canals. Thus, for middle-upper thoracic pedicle screw placement, a more individualized technique is needed to improve accuracy. The current advance technique is the computer-aided technologies [29], such as digitalized medical imaging simulation and digitalized navigation equipment. Nevertheless, the price factor severely limits the application of computer-aided technologies (especially for navigation equipment).

Taking the example of the Mimics 14.0 3D image processing software used in this study, the system requires the ability to handle the complex software, as well as the necessity to record the measured parameters and simulate it in mind, which makes it prone to disagreement, and especially most prone to bias in determining the entry points. Nevertheless, 1:1 printed model is intuitive and convenient. The information can be directly obtained from the model, and preoperative simulations can also be performed on the model, which can be used for comparison and reference during the surgery, which is the greatest significance of this study. As described above, the middle-upper thoracic vertebrae surgery has a steep learning curve. The 3-D printing model offered a perfect tool for the doctor to practice, try their hand and gather experience.

The 3D printing technique used in this study is relatively inexpensive. At present, a printer cheaper than 10,000 RMB (1458 US-dollar) is sufficient to meet the requirement. Also, many third-party companies offered printing services. They only need image data anonymously and a low price (around 1000 RMB or 146 US-dollar) to print out any form of solid models needed.

The advantages of the 3D printing technology base on the seven patients series is as follows: (1) Surgeons usually construct 3D images in the brain by reading 2D images to predict the difficulty of the surgery. These surgeon-dependent experience and judgment are prone to disagreement for difficult surgeries. However, the 1:1 3D solid model provides the visual aid with significant resemblance to the surgery object, which enables to eliminate individual differences in reading 2D images. (2) For patients with difficulty in placement of middle-upper thoracic pedicle screw, especially for those requiring long-segment screw placement, the secure and effective pedicle screws can be directly selected based on the 1:1 model, preventing subsequent complications caused by errors in selecting the screw diameter or length. (3) Sterilized models can be placed in the sterile surgical area, and surgeons can directly refer to the entry point, inclined angle, head-tilting or tail-tilting angles, and other important parameters to improve the accuracy of bare-hands screw placement. (4) The economically affordable method allows all levels of hospitals to adopt this technology, which is suitable for implementation in hospitals of different levels. (5) It may diminish the learning curve of young doctors and enable them to establish confidence in patients with spinal pedicle screw placement difficulty.

Of course, like any other technology, there are also disadvantages of this method: (1) the commonly used resin material is not heat resistant, which tends to deform under high temperature. Thus, it cannot withstand medical high-temperature sterilization and requires an ethylene oxide fumigation technology. (2) If the third party entrusted to print the model had no medical background, they may not able to provide adequate finished reconstruction model and suitably satisfied a professional point of view, and often require cooperation with medical staff for a certain period. (3) Anonymity should be maintained in entrusting printing, so as to protect patients’ privacy.

This study still has many shortcomings. Only a small number of patients were included due to relatively rare cases of trauma in the middle-upper thoracic “watershed” area. The limitations to this study includes the lack of control group and statistic evaluation. CT-based navigation system should be a suitable control group for this study, however such system is beyond our institutional affordability.

Before the assistance of 3-D printing, the average operation time of middle-upper thoracic trauma in our institution is over 3–4 h. Some patients have to switch to pedicle hooks due to screw misplacement. Therefore, it is difficult for us to set a control group without 3-D printing. In this study, our aim is to share some preliminary experience. The benefits of 3-D printing needs to be further measured by larger RCT studies with adequate control.

## Conclusion

3D printing technique is an intuitive and effective assistive technology in pedicle screw fixation for treating middle-upper thoracic vertebra, which can improve the accuracy of bare-hands screw placement and reduce empirical errors.

## Additional files

Additional file 1: Typical case 1. Fig. S9. A 48-year-old female suffering from traffic injury. Case1. **Fig. S10.** Thoracic anterioposterior X-ray revealed damage of T7 and T8 pedicle morphology as well as lateral displacement. Case1. **Fig. S11.** CT reconstruction confirmed T7 and T8 blowout fracture of type C. Case1. **Fig. S12.** Magnetic resonance imaging showed upper thoracic kyphosis and spinal cord compression. Case1. **Fig. S13.** Upper wall of the right T6 pedicle was pierced accidentally, and then the pedicle screw could not be satisfactorily placed even by the repeated operation. Case1. **Fig. S14.** Intraoperative observation of the model showed intact left pedicle in the diseased T7. On the basis of the model, screws with a diameter of 4.5 mm and length 35 mm were selected and placed in the diseased vertebra. Case1. **Fig. S15.** Postoperative anteroposterior X-ray prompted correction of lateral displacement of the middle-upper thoracic vertebrae. Case1. **Fig. S16.** Postoperative lateral X-ray prompted good placement of pedicle screws. Case1. **Fig. S17.** Pedicle screw distribution was of level 0 at right T5, and of level 1 at left T5. Case1. **Fig. S18.** Pedicle screw distribution was of level 0 at right T6. Case1. **Fig. S19.** Pedicle screw distribution was of level 3 at the left side of the diseased T7, piercing the lateral wall, without causing adverse consequences. Case1. (ZIP 14627 kb)

Additional file 2: Typical case 2. Fig. S20. 62 years-old male, falling injury with T2/3 fracture type B1. Case 2. **Fig. S21.** Continuously printed 3D spine model for patient of Case2. **Fig. S22.** Individually printed 3D spine model for patient of Case2. **Fig. S23.** Postoperative anteroposterior X-ray showed correction fixation from T1 to T4 of the thoracic vertebrae. Case2. **Fig. S24.** Pedicle screw distribution was of level 1 at right T1, and of level 0 at left T1. Case2. **Fig. S25.** Pedicle screw distribution was of level 1 at right T2, and of level 0 at left T2. Case2. **Fig. S26.** Pedicle screw distribution was of level 0 at T3. Case2. **Fig. S27.** Pedicle screw distribution was of level 0 at T4. Case2. (ZIP 3450 kb)

## Abbreviations

ASIA: American Spinal Injury Association
CAD: Computer-aided design
CT: Computed tomography
