# Supplementary figures and images for: 3D printing–assisted preoperative plan of pedicle screw placement for middle-upper thoracic trauma: a cohort study
Source: BMC Musculoskelet Disord. 2017 Aug 11;18:348. doi: 10.1186/s12891-017-1703-1 (PMC5553797; doi:10.1186/s12891-017-1703-1)

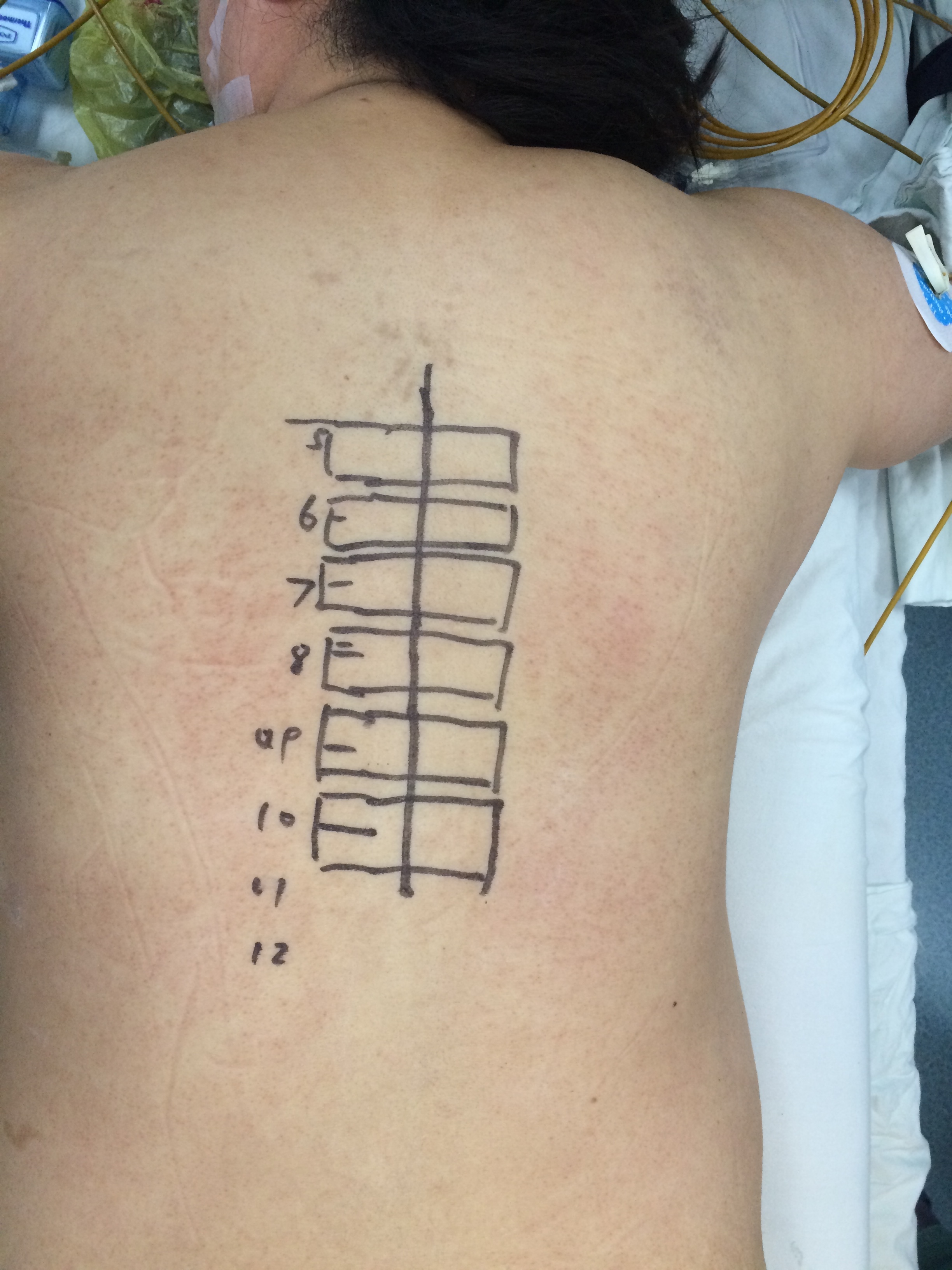

Supplement: Supplementary file 1 — A 48-year-old female suffering from traffic injury. Case1. Fig. S10. Thoracic anterioposterior X-ray revealed damage of T7 and T8 pedicle morphology as well as lateral displacement. Case1. Fig. S11. CT reconstruction confirmed T7 and T8 blowout fracture of type C. Case1. Fig. S12. Magnetic resonance imaging showed upper thoracic kyphosis and spinal cord compression. Case1. Fig. S13. Upper wall of the right T6 pedicle was pierced accidentally, and then the pedicle screw could not be satisfactorily placed even by the repeated operation. Case1. Fig. S14. Intraoperative observation of the model showed intact left pedicle in the diseased T7. On the basis of the model, screws with a diameter of 4.5 mm and length 35 mm were selected and placed in the diseased vertebra. Case1. Fig. S15. Postoperative anteroposterior X-ray prompted correction of lateral displacement of the middle-upper thoracic vertebrae. Case1. Fig. S16. Postoperative lateral X-ray prompted good placement of pedicle screws. Case1. Fig. S17. Pedicle screw distribution was of level 0 at right T5, and of level 1 at left T5. Case1. Fig. S18. Pedicle screw distribution was of level 0 at right T6. Case1. Fig. S19. Pedicle screw distribution was of level 3 at the left side of the diseased T7, piercing the lateral wall, without causing adverse consequences. Case1. (ZIP 14627 kb) [file 12891_2017_1703_MOESM1_ESM.zip › 9.jpg]

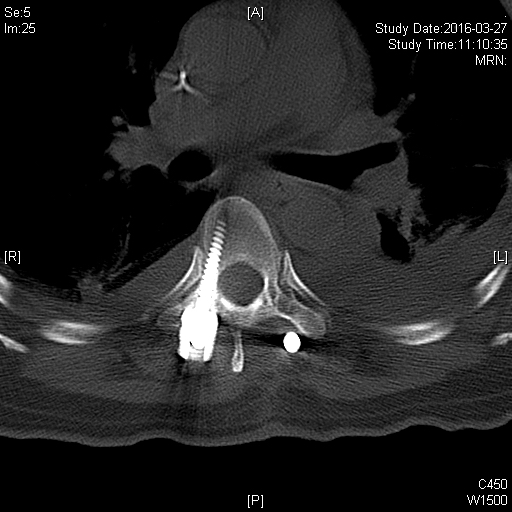

Supplement: Supplementary file 1 — A 48-year-old female suffering from traffic injury. Case1. Fig. S10. Thoracic anterioposterior X-ray revealed damage of T7 and T8 pedicle morphology as well as lateral displacement. Case1. Fig. S11. CT reconstruction confirmed T7 and T8 blowout fracture of type C. Case1. Fig. S12. Magnetic resonance imaging showed upper thoracic kyphosis and spinal cord compression. Case1. Fig. S13. Upper wall of the right T6 pedicle was pierced accidentally, and then the pedicle screw could not be satisfactorily placed even by the repeated operation. Case1. Fig. S14. Intraoperative observation of the model showed intact left pedicle in the diseased T7. On the basis of the model, screws with a diameter of 4.5 mm and length 35 mm were selected and placed in the diseased vertebra. Case1. Fig. S15. Postoperative anteroposterior X-ray prompted correction of lateral displacement of the middle-upper thoracic vertebrae. Case1. Fig. S16. Postoperative lateral X-ray prompted good placement of pedicle screws. Case1. Fig. S17. Pedicle screw distribution was of level 0 at right T5, and of level 1 at left T5. Case1. Fig. S18. Pedicle screw distribution was of level 0 at right T6. Case1. Fig. S19. Pedicle screw distribution was of level 3 at the left side of the diseased T7, piercing the lateral wall, without causing adverse consequences. Case1. (ZIP 14627 kb) [file 12891_2017_1703_MOESM1_ESM.zip › 18R6.png]

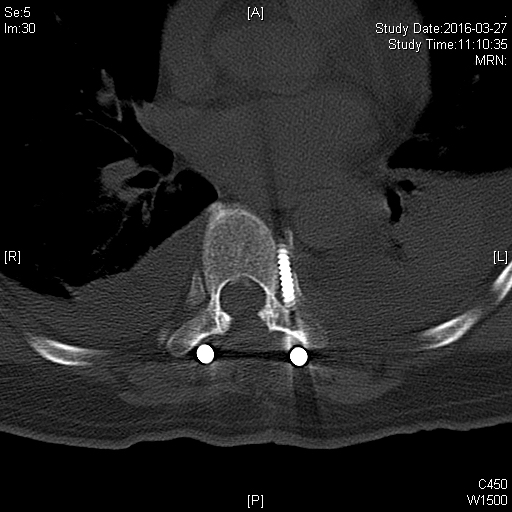

Supplement: Supplementary file 1 — A 48-year-old female suffering from traffic injury. Case1. Fig. S10. Thoracic anterioposterior X-ray revealed damage of T7 and T8 pedicle morphology as well as lateral displacement. Case1. Fig. S11. CT reconstruction confirmed T7 and T8 blowout fracture of type C. Case1. Fig. S12. Magnetic resonance imaging showed upper thoracic kyphosis and spinal cord compression. Case1. Fig. S13. Upper wall of the right T6 pedicle was pierced accidentally, and then the pedicle screw could not be satisfactorily placed even by the repeated operation. Case1. Fig. S14. Intraoperative observation of the model showed intact left pedicle in the diseased T7. On the basis of the model, screws with a diameter of 4.5 mm and length 35 mm were selected and placed in the diseased vertebra. Case1. Fig. S15. Postoperative anteroposterior X-ray prompted correction of lateral displacement of the middle-upper thoracic vertebrae. Case1. Fig. S16. Postoperative lateral X-ray prompted good placement of pedicle screws. Case1. Fig. S17. Pedicle screw distribution was of level 0 at right T5, and of level 1 at left T5. Case1. Fig. S18. Pedicle screw distribution was of level 0 at right T6. Case1. Fig. S19. Pedicle screw distribution was of level 3 at the left side of the diseased T7, piercing the lateral wall, without causing adverse consequences. Case1. (ZIP 14627 kb) [file 12891_2017_1703_MOESM1_ESM.zip › 19R6.png]

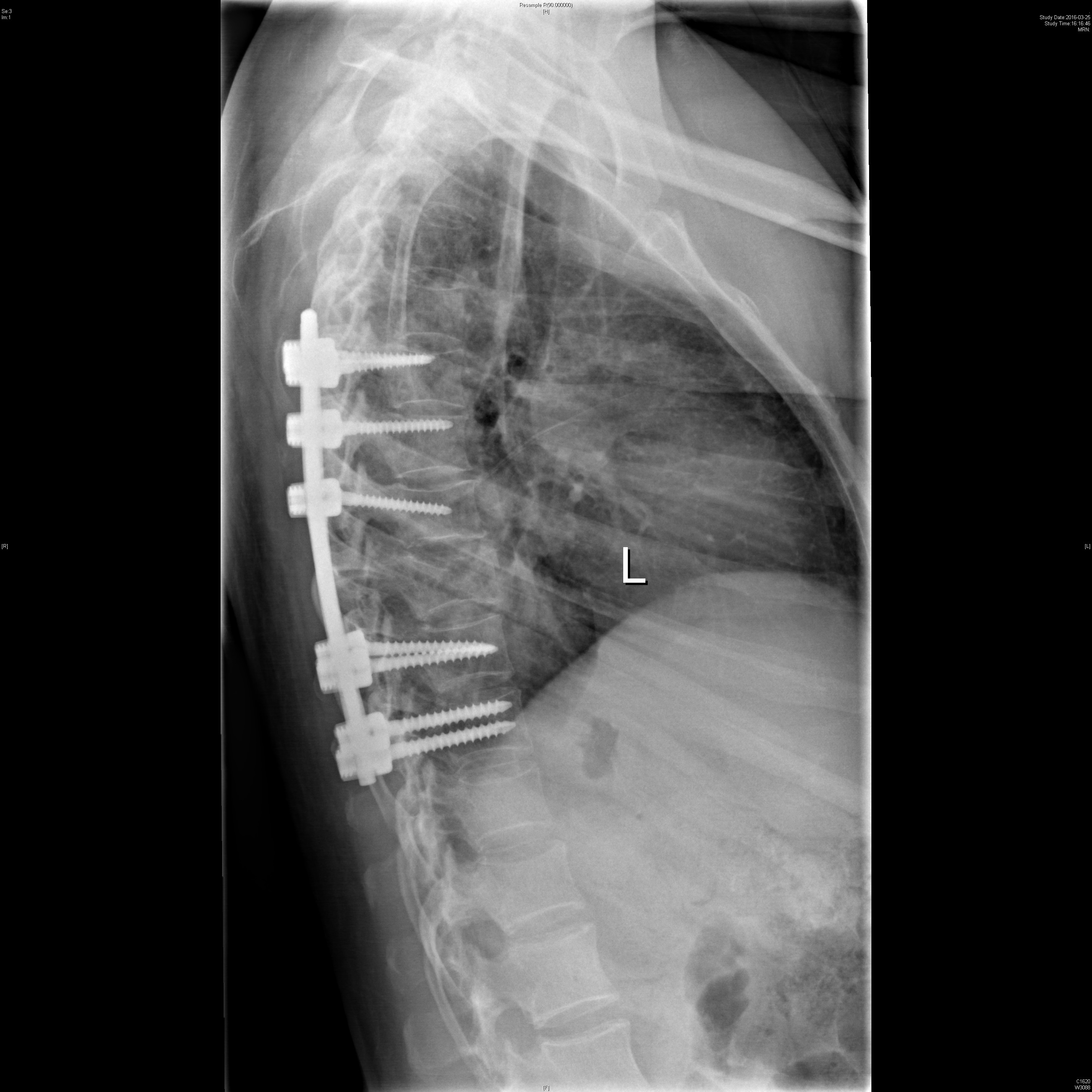

Supplement: Supplementary file 1 — A 48-year-old female suffering from traffic injury. Case1. Fig. S10. Thoracic anterioposterior X-ray revealed damage of T7 and T8 pedicle morphology as well as lateral displacement. Case1. Fig. S11. CT reconstruction confirmed T7 and T8 blowout fracture of type C. Case1. Fig. S12. Magnetic resonance imaging showed upper thoracic kyphosis and spinal cord compression. Case1. Fig. S13. Upper wall of the right T6 pedicle was pierced accidentally, and then the pedicle screw could not be satisfactorily placed even by the repeated operation. Case1. Fig. S14. Intraoperative observation of the model showed intact left pedicle in the diseased T7. On the basis of the model, screws with a diameter of 4.5 mm and length 35 mm were selected and placed in the diseased vertebra. Case1. Fig. S15. Postoperative anteroposterior X-ray prompted correction of lateral displacement of the middle-upper thoracic vertebrae. Case1. Fig. S16. Postoperative lateral X-ray prompted good placement of pedicle screws. Case1. Fig. S17. Pedicle screw distribution was of level 0 at right T5, and of level 1 at left T5. Case1. Fig. S18. Pedicle screw distribution was of level 0 at right T6. Case1. Fig. S19. Pedicle screw distribution was of level 3 at the left side of the diseased T7, piercing the lateral wall, without causing adverse consequences. Case1. (ZIP 14627 kb) [file 12891_2017_1703_MOESM1_ESM.zip › 16R6.png]

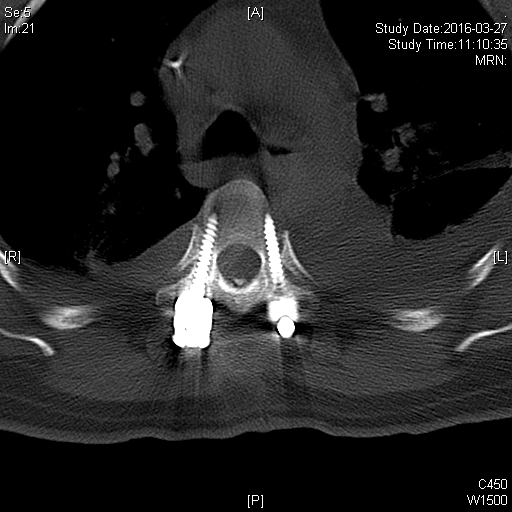

Supplement: Supplementary file 1 — A 48-year-old female suffering from traffic injury. Case1. Fig. S10. Thoracic anterioposterior X-ray revealed damage of T7 and T8 pedicle morphology as well as lateral displacement. Case1. Fig. S11. CT reconstruction confirmed T7 and T8 blowout fracture of type C. Case1. Fig. S12. Magnetic resonance imaging showed upper thoracic kyphosis and spinal cord compression. Case1. Fig. S13. Upper wall of the right T6 pedicle was pierced accidentally, and then the pedicle screw could not be satisfactorily placed even by the repeated operation. Case1. Fig. S14. Intraoperative observation of the model showed intact left pedicle in the diseased T7. On the basis of the model, screws with a diameter of 4.5 mm and length 35 mm were selected and placed in the diseased vertebra. Case1. Fig. S15. Postoperative anteroposterior X-ray prompted correction of lateral displacement of the middle-upper thoracic vertebrae. Case1. Fig. S16. Postoperative lateral X-ray prompted good placement of pedicle screws. Case1. Fig. S17. Pedicle screw distribution was of level 0 at right T5, and of level 1 at left T5. Case1. Fig. S18. Pedicle screw distribution was of level 0 at right T6. Case1. Fig. S19. Pedicle screw distribution was of level 3 at the left side of the diseased T7, piercing the lateral wall, without causing adverse consequences. Case1. (ZIP 14627 kb) [file 12891_2017_1703_MOESM1_ESM.zip › 17R6.png]

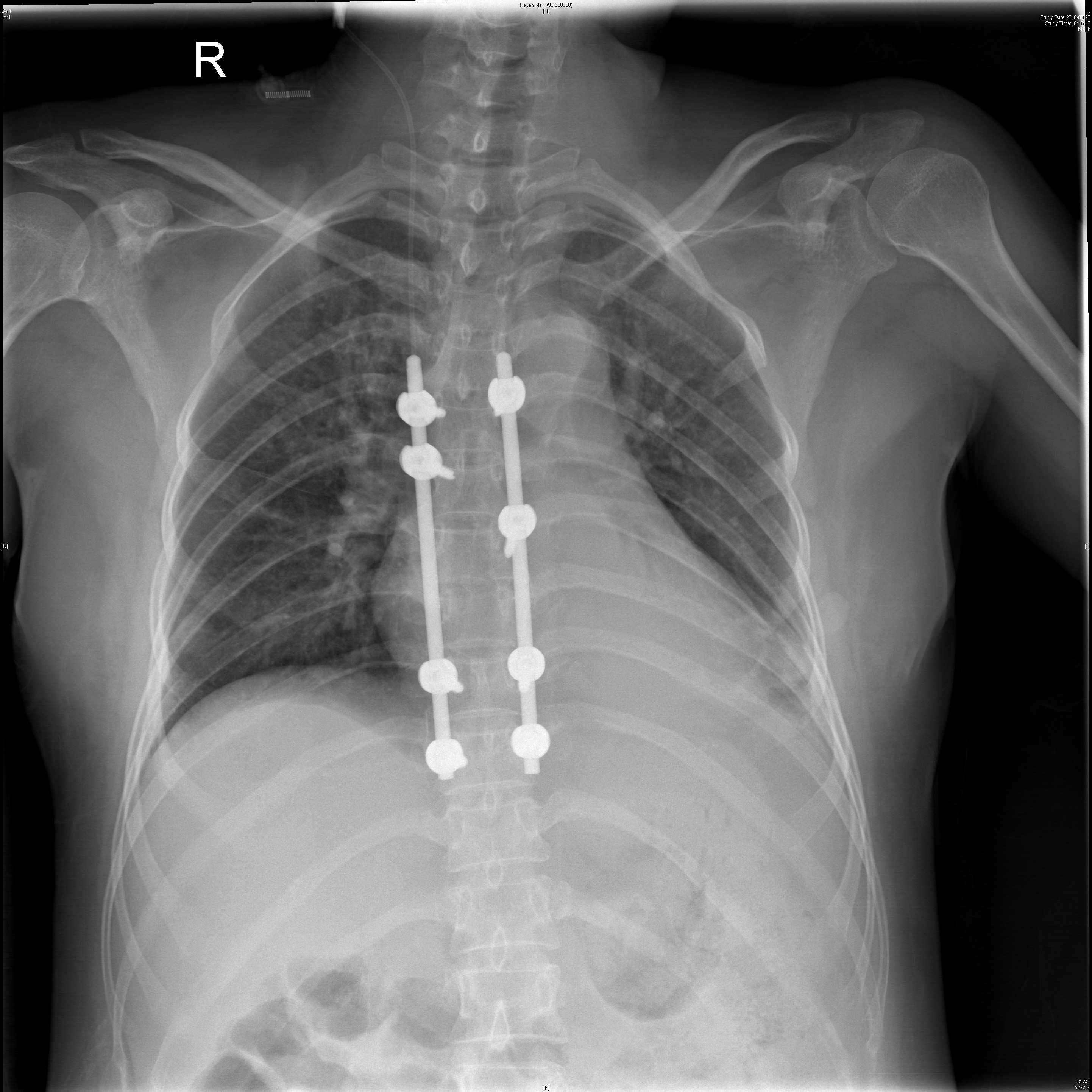

Supplement: Supplementary file 1 — A 48-year-old female suffering from traffic injury. Case1. Fig. S10. Thoracic anterioposterior X-ray revealed damage of T7 and T8 pedicle morphology as well as lateral displacement. Case1. Fig. S11. CT reconstruction confirmed T7 and T8 blowout fracture of type C. Case1. Fig. S12. Magnetic resonance imaging showed upper thoracic kyphosis and spinal cord compression. Case1. Fig. S13. Upper wall of the right T6 pedicle was pierced accidentally, and then the pedicle screw could not be satisfactorily placed even by the repeated operation. Case1. Fig. S14. Intraoperative observation of the model showed intact left pedicle in the diseased T7. On the basis of the model, screws with a diameter of 4.5 mm and length 35 mm were selected and placed in the diseased vertebra. Case1. Fig. S15. Postoperative anteroposterior X-ray prompted correction of lateral displacement of the middle-upper thoracic vertebrae. Case1. Fig. S16. Postoperative lateral X-ray prompted good placement of pedicle screws. Case1. Fig. S17. Pedicle screw distribution was of level 0 at right T5, and of level 1 at left T5. Case1. Fig. S18. Pedicle screw distribution was of level 0 at right T6. Case1. Fig. S19. Pedicle screw distribution was of level 3 at the left side of the diseased T7, piercing the lateral wall, without causing adverse consequences. Case1. (ZIP 14627 kb) [file 12891_2017_1703_MOESM1_ESM.zip › 15R6.png]

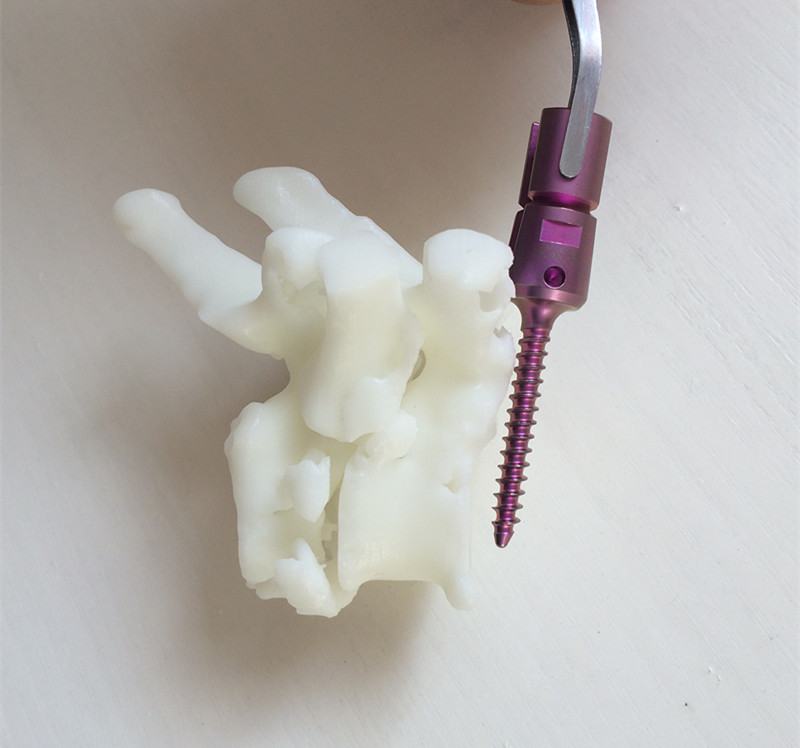

Supplement: Supplementary file 1 — A 48-year-old female suffering from traffic injury. Case1. Fig. S10. Thoracic anterioposterior X-ray revealed damage of T7 and T8 pedicle morphology as well as lateral displacement. Case1. Fig. S11. CT reconstruction confirmed T7 and T8 blowout fracture of type C. Case1. Fig. S12. Magnetic resonance imaging showed upper thoracic kyphosis and spinal cord compression. Case1. Fig. S13. Upper wall of the right T6 pedicle was pierced accidentally, and then the pedicle screw could not be satisfactorily placed even by the repeated operation. Case1. Fig. S14. Intraoperative observation of the model showed intact left pedicle in the diseased T7. On the basis of the model, screws with a diameter of 4.5 mm and length 35 mm were selected and placed in the diseased vertebra. Case1. Fig. S15. Postoperative anteroposterior X-ray prompted correction of lateral displacement of the middle-upper thoracic vertebrae. Case1. Fig. S16. Postoperative lateral X-ray prompted good placement of pedicle screws. Case1. Fig. S17. Pedicle screw distribution was of level 0 at right T5, and of level 1 at left T5. Case1. Fig. S18. Pedicle screw distribution was of level 0 at right T6. Case1. Fig. S19. Pedicle screw distribution was of level 3 at the left side of the diseased T7, piercing the lateral wall, without causing adverse consequences. Case1. (ZIP 14627 kb) [file 12891_2017_1703_MOESM1_ESM.zip › 14R6.jpg]

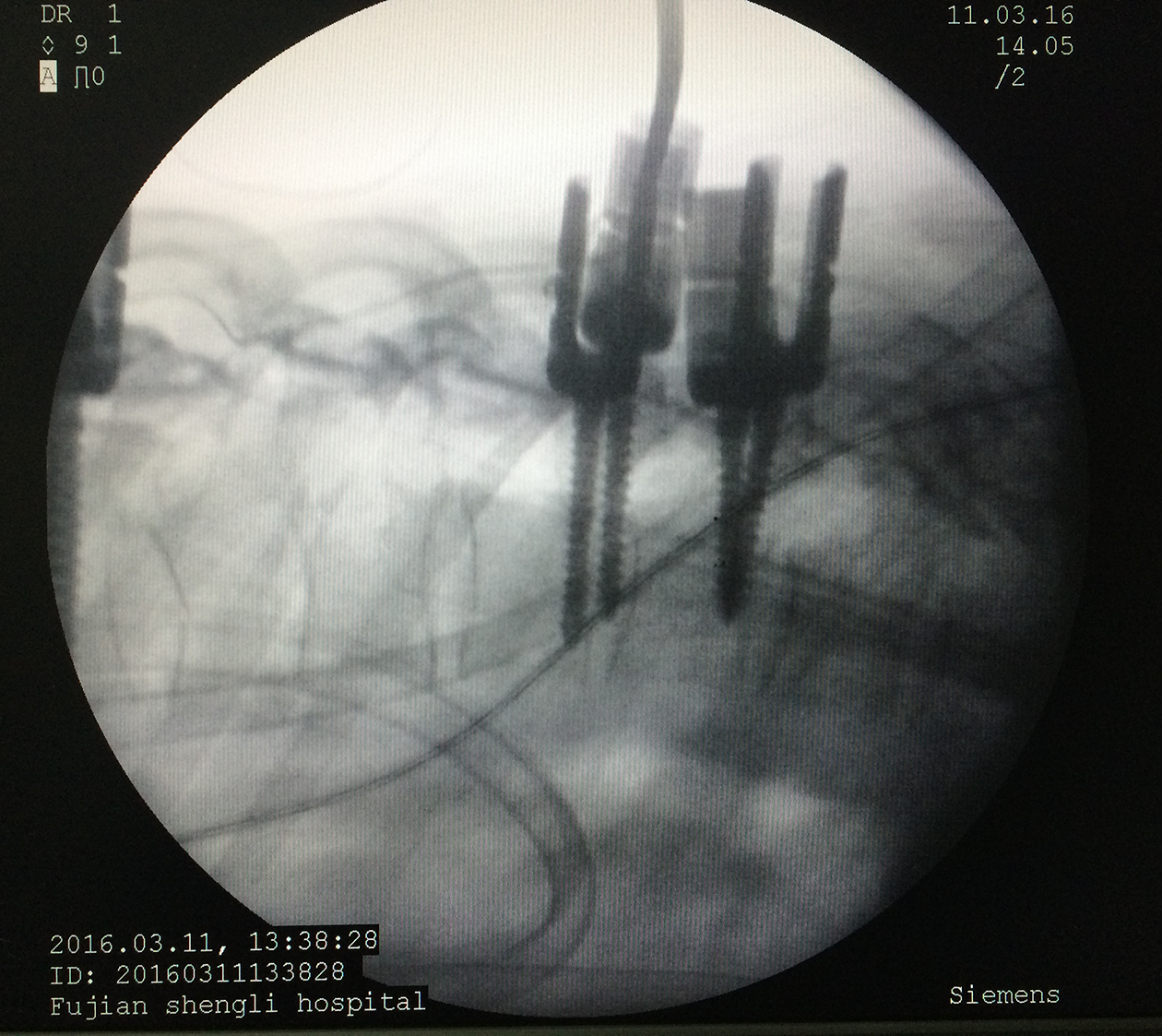

Supplement: Supplementary file 1 — A 48-year-old female suffering from traffic injury. Case1. Fig. S10. Thoracic anterioposterior X-ray revealed damage of T7 and T8 pedicle morphology as well as lateral displacement. Case1. Fig. S11. CT reconstruction confirmed T7 and T8 blowout fracture of type C. Case1. Fig. S12. Magnetic resonance imaging showed upper thoracic kyphosis and spinal cord compression. Case1. Fig. S13. Upper wall of the right T6 pedicle was pierced accidentally, and then the pedicle screw could not be satisfactorily placed even by the repeated operation. Case1. Fig. S14. Intraoperative observation of the model showed intact left pedicle in the diseased T7. On the basis of the model, screws with a diameter of 4.5 mm and length 35 mm were selected and placed in the diseased vertebra. Case1. Fig. S15. Postoperative anteroposterior X-ray prompted correction of lateral displacement of the middle-upper thoracic vertebrae. Case1. Fig. S16. Postoperative lateral X-ray prompted good placement of pedicle screws. Case1. Fig. S17. Pedicle screw distribution was of level 0 at right T5, and of level 1 at left T5. Case1. Fig. S18. Pedicle screw distribution was of level 0 at right T6. Case1. Fig. S19. Pedicle screw distribution was of level 3 at the left side of the diseased T7, piercing the lateral wall, without causing adverse consequences. Case1. (ZIP 14627 kb) [file 12891_2017_1703_MOESM1_ESM.zip › 13R6.tif]

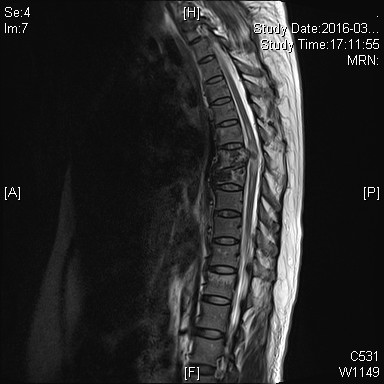

Supplement: Supplementary file 1 — A 48-year-old female suffering from traffic injury. Case1. Fig. S10. Thoracic anterioposterior X-ray revealed damage of T7 and T8 pedicle morphology as well as lateral displacement. Case1. Fig. S11. CT reconstruction confirmed T7 and T8 blowout fracture of type C. Case1. Fig. S12. Magnetic resonance imaging showed upper thoracic kyphosis and spinal cord compression. Case1. Fig. S13. Upper wall of the right T6 pedicle was pierced accidentally, and then the pedicle screw could not be satisfactorily placed even by the repeated operation. Case1. Fig. S14. Intraoperative observation of the model showed intact left pedicle in the diseased T7. On the basis of the model, screws with a diameter of 4.5 mm and length 35 mm were selected and placed in the diseased vertebra. Case1. Fig. S15. Postoperative anteroposterior X-ray prompted correction of lateral displacement of the middle-upper thoracic vertebrae. Case1. Fig. S16. Postoperative lateral X-ray prompted good placement of pedicle screws. Case1. Fig. S17. Pedicle screw distribution was of level 0 at right T5, and of level 1 at left T5. Case1. Fig. S18. Pedicle screw distribution was of level 0 at right T6. Case1. Fig. S19. Pedicle screw distribution was of level 3 at the left side of the diseased T7, piercing the lateral wall, without causing adverse consequences. Case1. (ZIP 14627 kb) [file 12891_2017_1703_MOESM1_ESM.zip › 12R6.png]

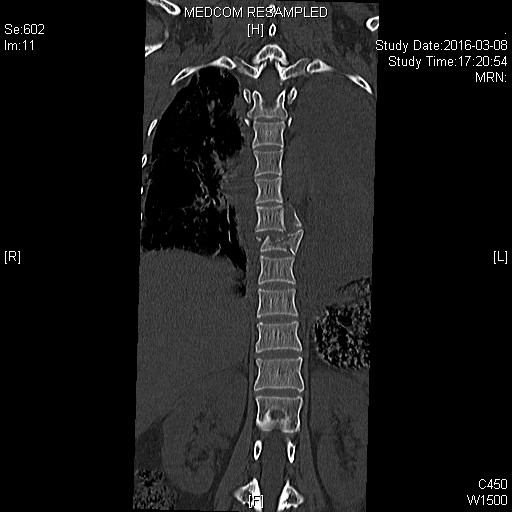

Supplement: Supplementary file 1 — A 48-year-old female suffering from traffic injury. Case1. Fig. S10. Thoracic anterioposterior X-ray revealed damage of T7 and T8 pedicle morphology as well as lateral displacement. Case1. Fig. S11. CT reconstruction confirmed T7 and T8 blowout fracture of type C. Case1. Fig. S12. Magnetic resonance imaging showed upper thoracic kyphosis and spinal cord compression. Case1. Fig. S13. Upper wall of the right T6 pedicle was pierced accidentally, and then the pedicle screw could not be satisfactorily placed even by the repeated operation. Case1. Fig. S14. Intraoperative observation of the model showed intact left pedicle in the diseased T7. On the basis of the model, screws with a diameter of 4.5 mm and length 35 mm were selected and placed in the diseased vertebra. Case1. Fig. S15. Postoperative anteroposterior X-ray prompted correction of lateral displacement of the middle-upper thoracic vertebrae. Case1. Fig. S16. Postoperative lateral X-ray prompted good placement of pedicle screws. Case1. Fig. S17. Pedicle screw distribution was of level 0 at right T5, and of level 1 at left T5. Case1. Fig. S18. Pedicle screw distribution was of level 0 at right T6. Case1. Fig. S19. Pedicle screw distribution was of level 3 at the left side of the diseased T7, piercing the lateral wall, without causing adverse consequences. Case1. (ZIP 14627 kb) [file 12891_2017_1703_MOESM1_ESM.zip › 11R6.png]

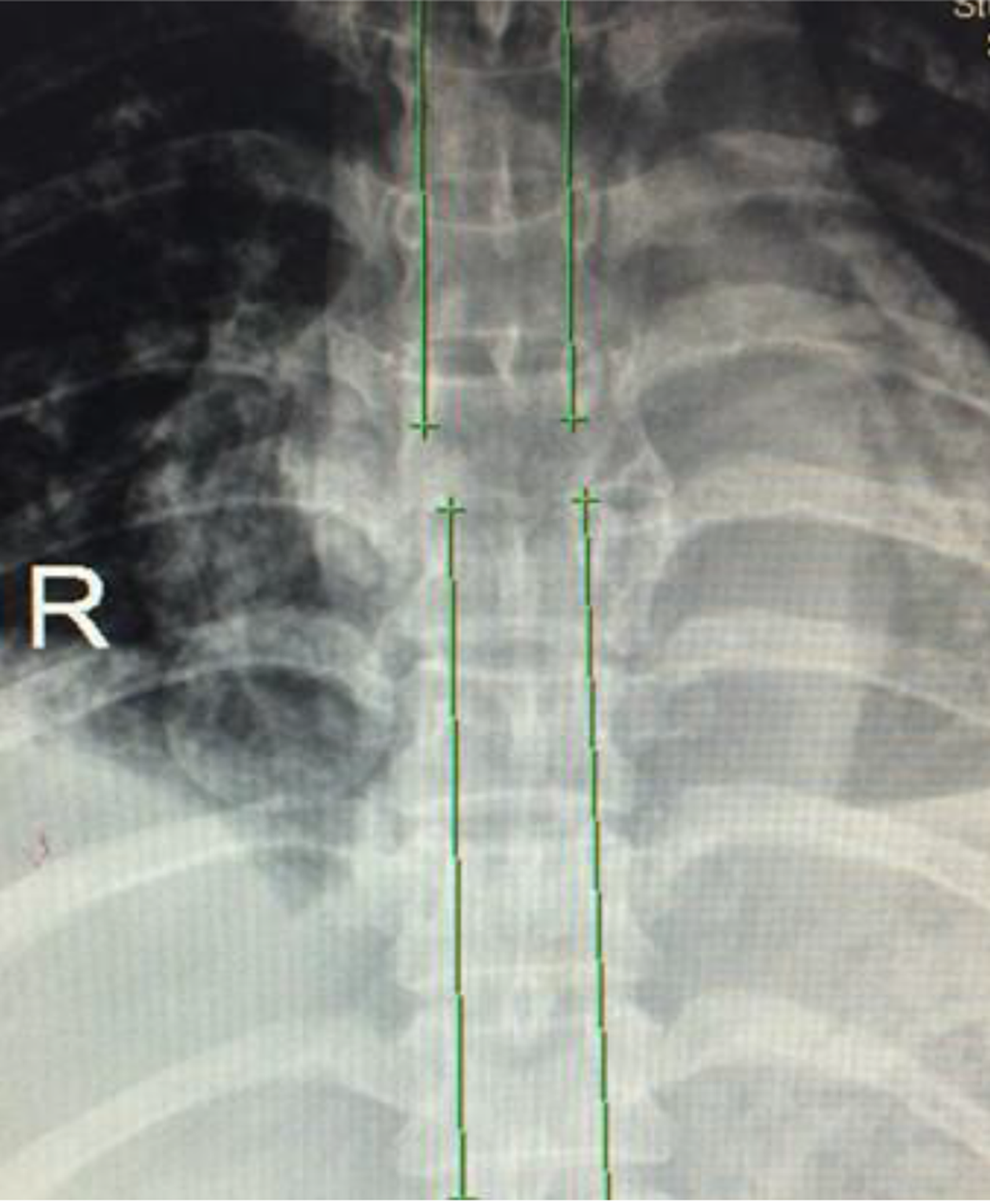

Supplement: Supplementary file 1 — A 48-year-old female suffering from traffic injury. Case1. Fig. S10. Thoracic anterioposterior X-ray revealed damage of T7 and T8 pedicle morphology as well as lateral displacement. Case1. Fig. S11. CT reconstruction confirmed T7 and T8 blowout fracture of type C. Case1. Fig. S12. Magnetic resonance imaging showed upper thoracic kyphosis and spinal cord compression. Case1. Fig. S13. Upper wall of the right T6 pedicle was pierced accidentally, and then the pedicle screw could not be satisfactorily placed even by the repeated operation. Case1. Fig. S14. Intraoperative observation of the model showed intact left pedicle in the diseased T7. On the basis of the model, screws with a diameter of 4.5 mm and length 35 mm were selected and placed in the diseased vertebra. Case1. Fig. S15. Postoperative anteroposterior X-ray prompted correction of lateral displacement of the middle-upper thoracic vertebrae. Case1. Fig. S16. Postoperative lateral X-ray prompted good placement of pedicle screws. Case1. Fig. S17. Pedicle screw distribution was of level 0 at right T5, and of level 1 at left T5. Case1. Fig. S18. Pedicle screw distribution was of level 0 at right T6. Case1. Fig. S19. Pedicle screw distribution was of level 3 at the left side of the diseased T7, piercing the lateral wall, without causing adverse consequences. Case1. (ZIP 14627 kb) [file 12891_2017_1703_MOESM1_ESM.zip › 10R6.tif]

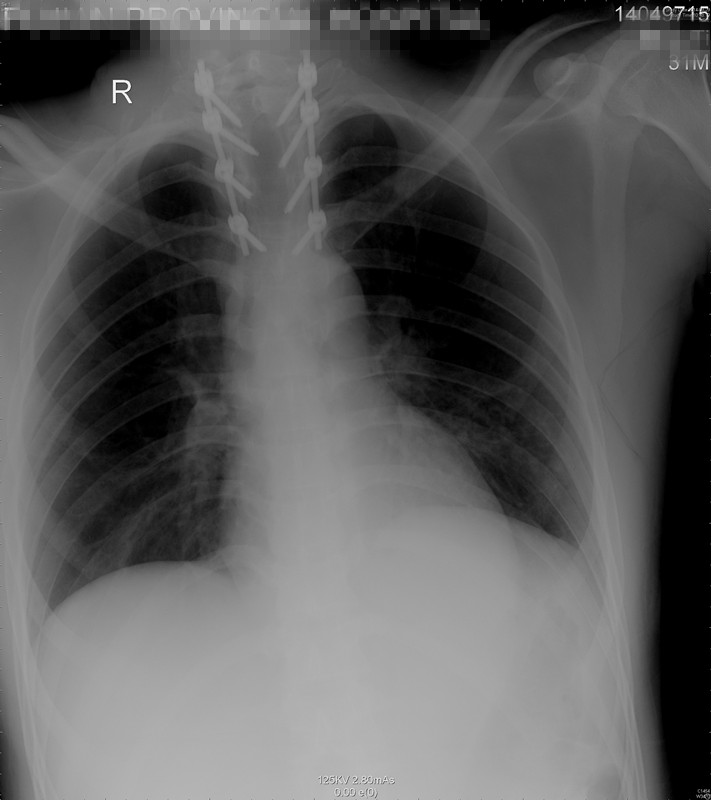

Supplement: Supplementary file 2 — 62 years-old male, falling injury with T2/3 fracture type B1. Case 2. Fig. S21. Continuously printed 3D spine model for patient of Case2. Fig. S22. Individually printed 3D spine model for patient of Case2. Fig. S23. Postoperative anteroposterior X-ray showed correction fixation from T1 to T4 of the thoracic vertebrae. Case2. Fig. S24. Pedicle screw distribution was of level 1 at right T1, and of level 0 at left T1. Case2. Fig. S25. Pedicle screw distribution was of level 1 at right T2, and of level 0 at left T2. Case2. Fig. S26. Pedicle screw distribution was of level 0 at T3. Case2. Fig. S27. Pedicle screw distribution was of level 0 at T4. Case2. (ZIP 3450 kb) [file 12891_2017_1703_MOESM2_ESM.zip › 23.png]

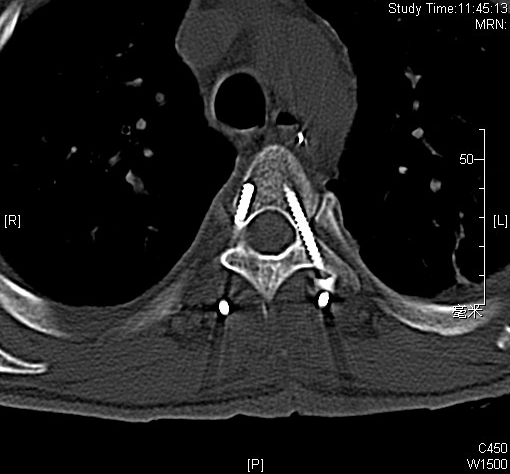

Supplement: Supplementary file 2 — 62 years-old male, falling injury with T2/3 fracture type B1. Case 2. Fig. S21. Continuously printed 3D spine model for patient of Case2. Fig. S22. Individually printed 3D spine model for patient of Case2. Fig. S23. Postoperative anteroposterior X-ray showed correction fixation from T1 to T4 of the thoracic vertebrae. Case2. Fig. S24. Pedicle screw distribution was of level 1 at right T1, and of level 0 at left T1. Case2. Fig. S25. Pedicle screw distribution was of level 1 at right T2, and of level 0 at left T2. Case2. Fig. S26. Pedicle screw distribution was of level 0 at T3. Case2. Fig. S27. Pedicle screw distribution was of level 0 at T4. Case2. (ZIP 3450 kb) [file 12891_2017_1703_MOESM2_ESM.zip › 27R6.png]

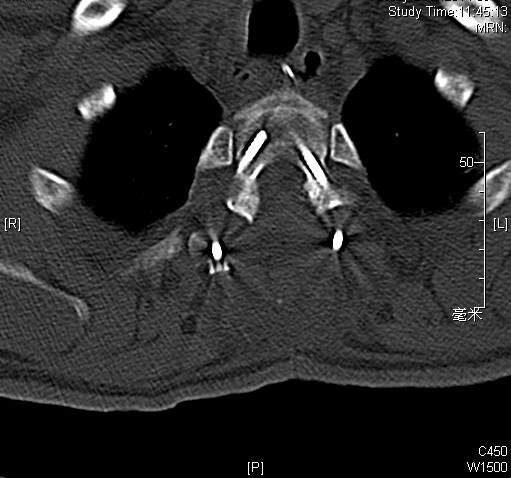

Supplement: Supplementary file 2 — 62 years-old male, falling injury with T2/3 fracture type B1. Case 2. Fig. S21. Continuously printed 3D spine model for patient of Case2. Fig. S22. Individually printed 3D spine model for patient of Case2. Fig. S23. Postoperative anteroposterior X-ray showed correction fixation from T1 to T4 of the thoracic vertebrae. Case2. Fig. S24. Pedicle screw distribution was of level 1 at right T1, and of level 0 at left T1. Case2. Fig. S25. Pedicle screw distribution was of level 1 at right T2, and of level 0 at left T2. Case2. Fig. S26. Pedicle screw distribution was of level 0 at T3. Case2. Fig. S27. Pedicle screw distribution was of level 0 at T4. Case2. (ZIP 3450 kb) [file 12891_2017_1703_MOESM2_ESM.zip › 25R6.png]

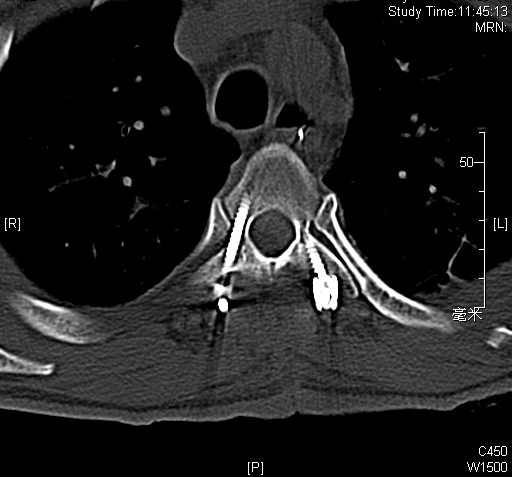

Supplement: Supplementary file 2 — 62 years-old male, falling injury with T2/3 fracture type B1. Case 2. Fig. S21. Continuously printed 3D spine model for patient of Case2. Fig. S22. Individually printed 3D spine model for patient of Case2. Fig. S23. Postoperative anteroposterior X-ray showed correction fixation from T1 to T4 of the thoracic vertebrae. Case2. Fig. S24. Pedicle screw distribution was of level 1 at right T1, and of level 0 at left T1. Case2. Fig. S25. Pedicle screw distribution was of level 1 at right T2, and of level 0 at left T2. Case2. Fig. S26. Pedicle screw distribution was of level 0 at T3. Case2. Fig. S27. Pedicle screw distribution was of level 0 at T4. Case2. (ZIP 3450 kb) [file 12891_2017_1703_MOESM2_ESM.zip › 26R6.png]

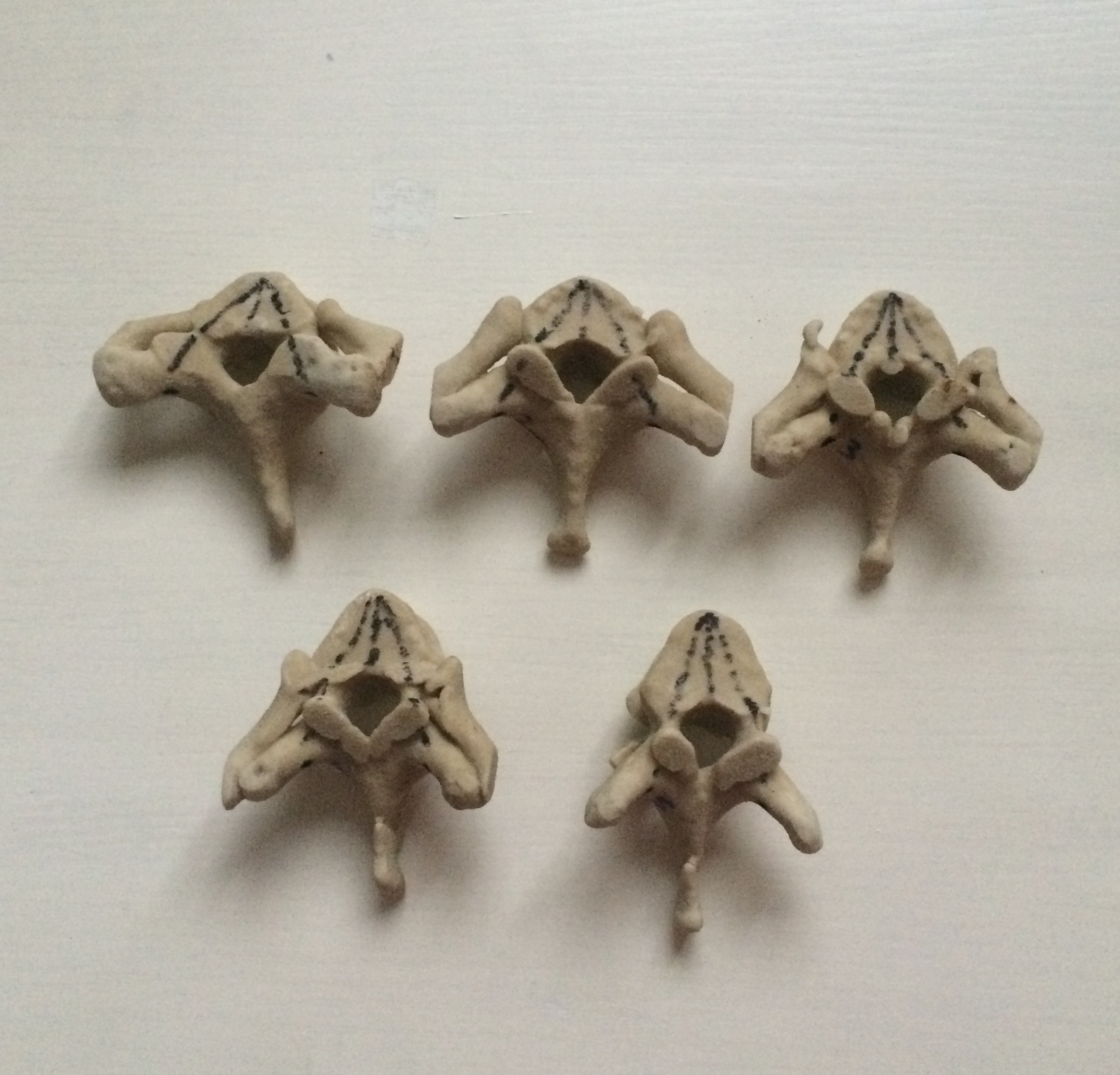

Supplement: Supplementary file 2 — 62 years-old male, falling injury with T2/3 fracture type B1. Case 2. Fig. S21. Continuously printed 3D spine model for patient of Case2. Fig. S22. Individually printed 3D spine model for patient of Case2. Fig. S23. Postoperative anteroposterior X-ray showed correction fixation from T1 to T4 of the thoracic vertebrae. Case2. Fig. S24. Pedicle screw distribution was of level 1 at right T1, and of level 0 at left T1. Case2. Fig. S25. Pedicle screw distribution was of level 1 at right T2, and of level 0 at left T2. Case2. Fig. S26. Pedicle screw distribution was of level 0 at T3. Case2. Fig. S27. Pedicle screw distribution was of level 0 at T4. Case2. (ZIP 3450 kb) [file 12891_2017_1703_MOESM2_ESM.zip › 22R6.jpg]

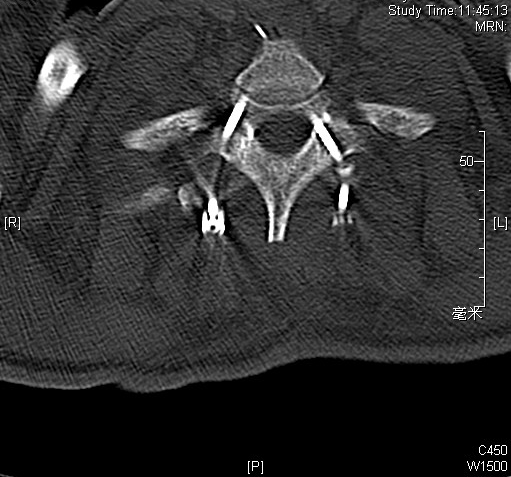

Supplement: Supplementary file 2 — 62 years-old male, falling injury with T2/3 fracture type B1. Case 2. Fig. S21. Continuously printed 3D spine model for patient of Case2. Fig. S22. Individually printed 3D spine model for patient of Case2. Fig. S23. Postoperative anteroposterior X-ray showed correction fixation from T1 to T4 of the thoracic vertebrae. Case2. Fig. S24. Pedicle screw distribution was of level 1 at right T1, and of level 0 at left T1. Case2. Fig. S25. Pedicle screw distribution was of level 1 at right T2, and of level 0 at left T2. Case2. Fig. S26. Pedicle screw distribution was of level 0 at T3. Case2. Fig. S27. Pedicle screw distribution was of level 0 at T4. Case2. (ZIP 3450 kb) [file 12891_2017_1703_MOESM2_ESM.zip › 24R6.png]

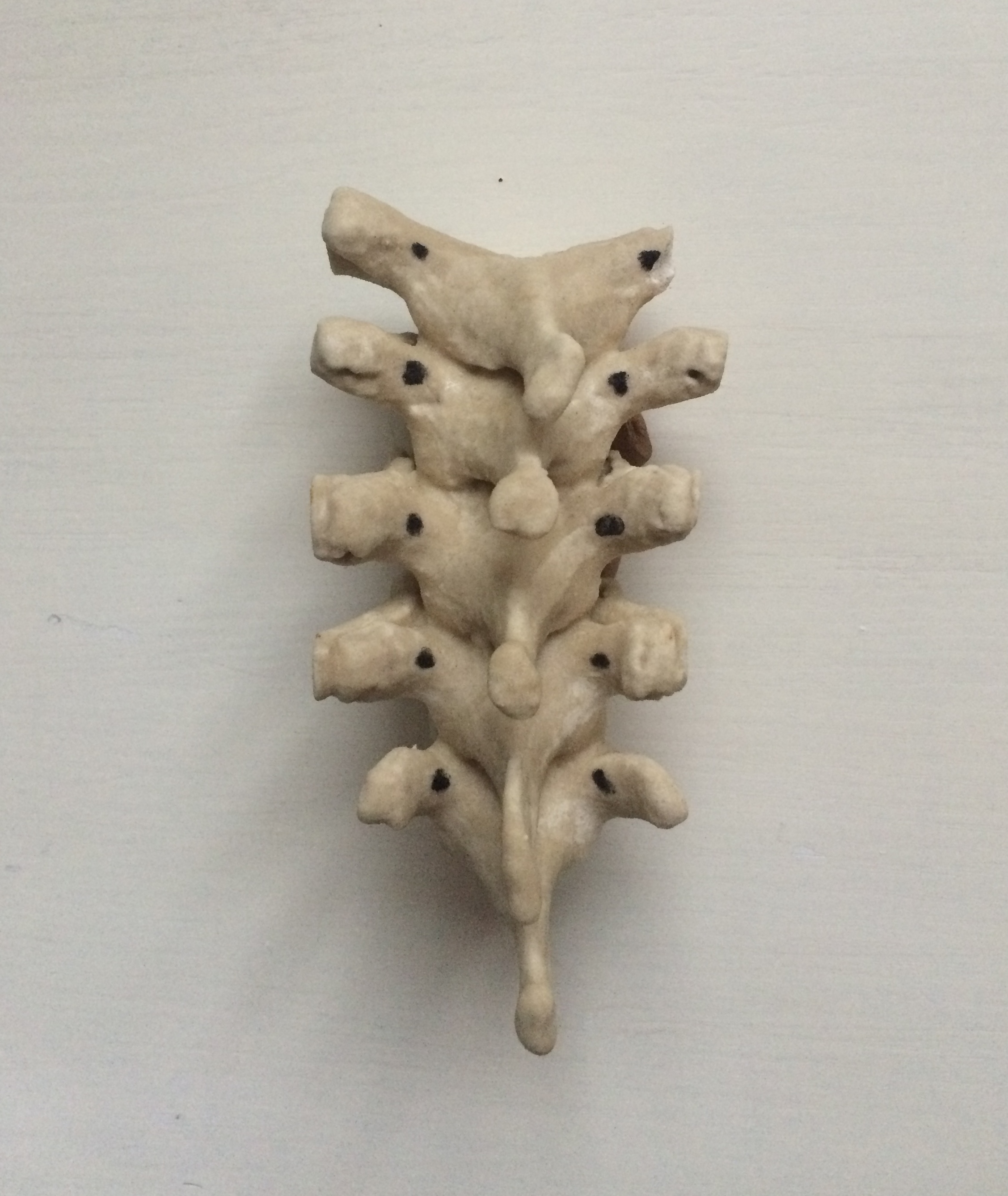

Supplement: Supplementary file 2 — 62 years-old male, falling injury with T2/3 fracture type B1. Case 2. Fig. S21. Continuously printed 3D spine model for patient of Case2. Fig. S22. Individually printed 3D spine model for patient of Case2. Fig. S23. Postoperative anteroposterior X-ray showed correction fixation from T1 to T4 of the thoracic vertebrae. Case2. Fig. S24. Pedicle screw distribution was of level 1 at right T1, and of level 0 at left T1. Case2. Fig. S25. Pedicle screw distribution was of level 1 at right T2, and of level 0 at left T2. Case2. Fig. S26. Pedicle screw distribution was of level 0 at T3. Case2. Fig. S27. Pedicle screw distribution was of level 0 at T4. Case2. (ZIP 3450 kb) [file 12891_2017_1703_MOESM2_ESM.zip › 21R6.jpg]

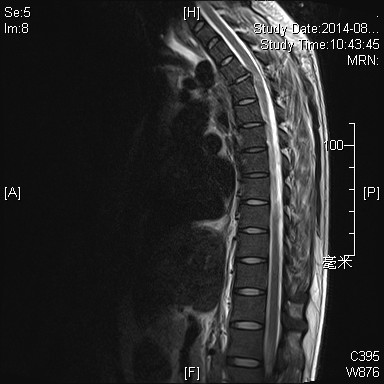

Supplement: Supplementary file 2 — 62 years-old male, falling injury with T2/3 fracture type B1. Case 2. Fig. S21. Continuously printed 3D spine model for patient of Case2. Fig. S22. Individually printed 3D spine model for patient of Case2. Fig. S23. Postoperative anteroposterior X-ray showed correction fixation from T1 to T4 of the thoracic vertebrae. Case2. Fig. S24. Pedicle screw distribution was of level 1 at right T1, and of level 0 at left T1. Case2. Fig. S25. Pedicle screw distribution was of level 1 at right T2, and of level 0 at left T2. Case2. Fig. S26. Pedicle screw distribution was of level 0 at T3. Case2. Fig. S27. Pedicle screw distribution was of level 0 at T4. Case2. (ZIP 3450 kb) [file 12891_2017_1703_MOESM2_ESM.zip › 20R6.png]
